# A Multi-Centre Randomized Controlled Trial Comparing Connective Tissue Graft with Collagen Matrix to Increase Buccal Soft Tissue Thickness: A Cone-Beam CT Analysis

**DOI:** 10.3390/jcm12082977

**Published:** 2023-04-19

**Authors:** Célien Eeckhout, Fauve Vuylsteke, Lorenz Seyssens, Véronique Christiaens, Thomas De Bruyckere, Aryan Eghbali, Stijn Vervaeke, Faris Younes, Jan Cosyn

**Affiliations:** 1 Department of Periodontology and Oral Implantology, Faculty of Medicine and Health Sciences, Oral Health Sciences, Ghent University, Corneel Heymanslaan 10, 9000 Ghent, Belgium; 2 Private Practice Ortho Paro Care, Mankevosstraat 5, 1860 Meise, Belgium

**Keywords:** connective tissue graft, collagen matrix, dental implant, single tooth, buccal bone, CBCT

## Abstract

(1) Aim: a cross-linked porcine-derived collagen matrix (CMX) has been developed for soft tissue augmentation. Although this grafting material does not require a second surgical site, recent findings have indicated deeper pockets, more marginal bone loss and more midfacial recession in the short term when compared to connective tissue graft (CTG). Hence, the aim of the present study was to evaluate the safety of CMX based on buccal bone loss over a one-year period. (2) Methods: Patients who were missing a single tooth in the anterior maxilla were included, in whom the failing tooth had been removed at least 3 months prior and who presented a horizontal mucosa defect. All sites had a bucco-palatal bone dimension of at least 6 mm as assessed on Cone-Beam Computed Tomography (CBCT) to ensure complete embedding of an implant by bone. All patients received a single implant and an immediate implant restoration using a full digital workflow. Sites were randomly allocated to the control (CTG) or test group (CMX) to increase buccal soft tissue thickness. All surgeries were performed by means of full thickness mucoperiosteal flap elevation, placing CTG and CMX in contact with the buccal bone wall. Safety was assessed by evaluating the impact of CTG and CMX on buccal bone loss over a one-year period using superimposed CBCT scans. (3) Results: thirty patients were included per group (control: 50% females, mean age 50; test: 53% females, mean age 48) and 51 (control: 25; test: 26) could be analyzed for buccal bone loss. At 1 mm apical to the implant-abutment interface (IAI), most horizontal resorption was found pointing to 0.44 mm in the control group and 0.59 mm in the test group. The difference of 0.14 mm (95% CI: −0.17–0.46) was not statistically significant (*p* = 0.366). At 3 mm and 5 mm apical to the IAI, the difference between the groups was 0.18 mm (95% CI: −0.05–0.40; *p* = 0.128) and 0.02 mm (95% CI: −0.24–0.28; *p* = 0.899), respectively. Vertical buccal bone loss amounted to 1.12 mm in the control group and 1.14 mm in the test group. The difference of 0.02 mm (95% CI: −0.53–0.49) was not statistically significant (*p* = 0.926). (4) Conclusions: In the short term, soft tissue augmentation with CTG or CMX results in limited buccal bone loss. CMX is a safe alternative to CTG. Longer follow-up is needed to assess the impact of soft tissue augmentation on buccal bone.

## 1. Introduction

Following tooth extraction, successive biological processes occur resulting in substantial shrinkage of the alveolar process [1]. This mainly develops in the horizontal dimension, leading to a loss of buccal convexity [2]. In the early days, implant therapy merely focused on the restoration of function, leaving such soft tissue defects usually untreated. Even though implants installed in healed bone without augmentation procedures demonstrate high survival rates and limited marginal bone loss in the long term [3], their aesthetic outcome in the anterior maxilla is usually poor [4]. Contemporary treatment includes more and more soft tissue grafting in order to re-establish buccal convexity. A recent systematic review on patient-reported outcomes showed that soft tissue grafting can improve patient satisfaction and aesthetics compared to non-grafted sites [5].

Connective tissue graft (CTG) is considered the gold standard in soft tissue augmentation at implant sites [6]. Ultrasonic assessment yielded high stability of CTG up to 5 years of function [7]. Recent RCTs using profilometric analyses demonstrated an increase in buccal soft tissue profile of 1 mm up to 3 years of function [8,9]. However, CTG requires a second surgical site at the lateral palate or tuberosity for graft harvesting, which could induce additional morbidity [10]. Also, the available tissue at these donor sites limits the amount of tissue that can be harvested. To eliminate these disadvantages of CTG, a cross-linked porcine-derived collagen matrix (Geistlich Fibro-Gide^®^, Geistlich Pharma AG, Wolhusen, Switzerland) (CMX) has been developed. In a multi-center RCT, CTG was compared with CMX to increase soft tissue thickness at the buccal aspect of single implants installed in healed bone [11]. At a one-year follow-up, CTG yielded an increase in buccal soft tissue profile of 0.98 mm, while CMX resulted in an increase of 0.57 mm. Both were found to be effective, although the difference between the groups was statistically significant.

Apart from effectiveness, safety is also important when evaluating treatment modalities. In that respect, CMX treated sites demonstrated deeper pockets, more marginal bone loss and more midfacial recession at 3 months follow-up when compared to CTG treated sites [12]. At a one-year follow-up, CMX treated sites still showed more marginal bone loss [11]. These findings suggest that CMX induces chronic inflammation in the adjacent tissues due to cross-linking. Cross-linking prevents rapid biodegradation, which has been shown to result in more soft tissue augmentation at 3 months follow-up than non-cross-linked porcine-derived collagen matrices [13,14]. On the contrary, cross-linking increases inflammation which potentially led to wound healing complications [15,16]. A preclinical study showed moderate inflammation was observed for up to 2 months when using CMX [17]. In humans, CMX seems to induce more inflammation and over a longer time period, as indicated by clinical parameters in a multi-center RCT [11,12]. Hence, the objective of the present study was to assess the safety of CMX by comparing buccal bone loss between CTG and CMX treated sites based on Cone-Beam Computed Tomography (CBCT) analyses. The following research hypotheses were selected:

**Null Hypothesis H_0_:**  *There is no difference in buccal bone boss between CTG and CMX.*

**Alternative Hypothesis H_1_:**  *There is a difference in buccal bone loss between CTG and CMX.*

## 2. Materials and Methods

### 2.1. Study Design and Patient Selection

The present study is based on the same patient population enrolled in a previous multi-centre RCT comparing CTG with CMX [12]. Patients requiring a single implant restoration in the premaxilla were recruited between September 2019 and September 2020 based on inclusion and exclusion criteria. 

Inclusion criteria were as follows:

- At least 21 years old;
- Proper oral hygiene, determined as full-mouth plaque score ≤25% [18];
- One missing tooth gap in the premaxilla (15–25) with both adjacent teeth present;
- Failing tooth extracted at least 3 months before enrollment;
- Not less than 5 mm of keratinized mucosa available at the level of the missing tooth;
- Horizontal mucosa defect at the missing tooth gap as clinically assessed (bucco-palatal loss of tissue with a normal apicocoronal crest height) [19];
- At least 6 mm of bone in bucco-palatal direction at the central and crestal aspect of the missing tooth, as evaluated on CBCT to ensure that the implant is completely surrounded by bone;
- Signed informed consent.

Exclusion criteria were as follows:

- Systemic diseases;
- Smoking;
- Periodontal disease;
- Untreated caries lesions;
- Horizontal bone augmentation required during implant placement.

The study was approved by the Ethical Committee of Ghent University Hospital (B670201940413) and registered in ClinicalTrials.gov (NCT04210596). It was conducted in accordance with the ethical standards of the Declaration of Helsinki in 1975, as revised in 2013. The study was reported following the guidelines of the CONSORT statement [20]. 

### 2.2. Randomization, Allocation Concealment and Blinding

Six experienced and calibrated implant surgeons working in different periodontal practices in Belgium were selected to collaborate in this multi-centre RCT. Patients were allocated at random to either the control group (CTG) or test group (CMX). Block randomization was applied per centre by providing an equal number of sealed envelopes for each treatment group. Group assignment was announced just after implant installation and remained concealed for the evaluating examiner and statistician to allow for unbiased registrations and analyses, respectively.

### 2.3. Treatment Procedures and Postoperative Care

Details on the treatment procedures and postoperative care can be found in an earlier paper [12]. In brief, a low-dose CBCT and intra-oral scan were taken and imported in designated software (DTX Studio^®^, Nobel Biocare AB, Göteborg, Sweden) to produce a stereolythographic surgical guide and screw-retained CAD/CAM provisional restoration (TempShell^®^, Nobel Biocare AB, Göteborg, Sweden).

On the day of surgery, a crestal incision at the level of the missing tooth and sulcular incisions at the adjacent teeth were made to raise a full thickness mucoperiosteal flap. The prepared surgical guide was used to place a dental implant (NobelReplace CC PMC^®^ TiUnite, Nobel Biocare AB, Göteborg, Sweden) in a correct three-dimensional position. Thereupon, a sealed envelope was opened, containing the allocation for either one of two treatment modalities:

- Control group: autogenous connective tissue graft (CTG)
- Test group: collagen matrix (CMX: Geistlich Fibro-Gide^®^, Geistlich Pharma AG, Wolhusen, Switzerland)

**Figure 1:**
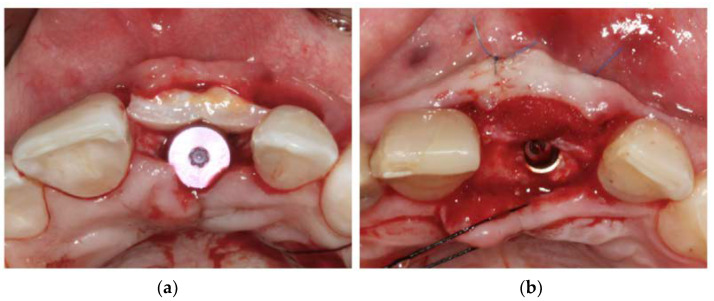
Clinical example of (**a**) connective tissue graft and (**b**) cross-linked porcine-derived col-lagen matrix.

In the control group, a CTG was harvested from the palatal mucosa in the premolar area by means of the single incision technique as described by De Bruyckere et al. [21]. The size of the CTG was adapted to the dimensions of the recipient site. Double-cross sutures (Vicryl^®^ Plus 4/0, Ethicon, Ohio, USA) were used to close the palatal wound. In the test group, a CMX was used, and dimensions were also matched to the defect. After releasing the muscle tension, the graft was placed onto the buccal bone wall and attached to the buccal mucosa with two single sutures (Seralon 6/0, Serag Wiessner, Naila, Germany). Figure 1 shows a clinical example of both soft tissue augmentation procedures.

Following the installation of the screw-retained provisional restoration (TempShell^®^, Nobel Biocare AB, Göteborg, Sweden), tension-free primary wound closure was achieved. Sutures were removed after 2 weeks. The provisional crown was replaced by a permanent crown by the general dentist after 3 months.

### 2.4. Horizontal Buccal Bone Loss

**Figure 2:**
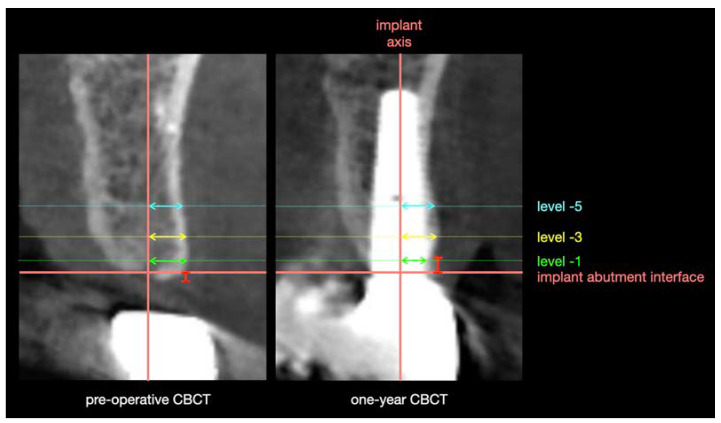
The following reference lines were constructed on the one-year CBCT in the center of the implant: the long axis of the implant, the level of the implant-abutment interface perpendicular to the long axis of the implant, level −1 mm, level −3 mm and level −5 mm apical to the implant-abutment interface and perpendicular to the long axis of the implant. Horizontal measurements were performed between the center of the implant and the buccal bone surface at the different levels (level −1 mm: green arrow, level −3 mm: yellow arrow, level −5 mm: blue arrow). Vertical measurement was performed between the implant-abutment interface and the buccal bone peak, parallel to the long axis of the implant (red arrow).

Horizontal buccal bone loss was assessed using the method of Seyssens et al. [22]. A low-dose CBCT was captured pre-operatively and at one-year follow-up. All CBCT images were taken using a ProMax 3D Max device (Planmeca, Helsinki, Finland) with the same standardized settings (90 kV, 6.3 mA, 9 seconds, voxel size 200 μm) and a same field of view 50 × 80 mm for each patient. Lip retractors were used to be able to properly identify the external soft tissue profile and, for the same reason, patients were asked to curl the tongue back. CBCTs from both time points were superimposed using specialized software (Invivo6, Osteoid Inc., Santa Clara, CA, USA). Figure 2 shows the measurement protocol. The following reference lines were constructed on the one-year CBCT in the center of the implant: the long axis of the implant, the level of the implant-abutment interface perpendicular to the long axis of the implant, level −1 mm, level −3 mm and level −5 mm apical to the implant-abutment interface and perpendicular to the long axis of the implant. Horizontal measurements were performed between the center of the implant and the buccal bone surface at the different levels. After completing all measurements on the one-year CBCT, the software was changed to the superimposed pre-operative image so that the same measurements could be done while all reference lines remained. Finally, horizontal buccal bone loss at −1 mm, −3 mm and −5 mm was calculated by subtracting one-year measurements from pre-operative measurements. 

### 2.5. Vertical Buccal Bone Loss

Vertical buccal bone loss was measured on superimposed CBCTs in the same software (Invivo6, Osteoid Inc., Santa Clara, CA, USA). Figure 2 shows the measurement protocol. First, the one-year vertical buccal bone level was measured between the implant-abutment interface and the buccal bone peak, parallel to the long axis of the implant. A negative value corresponded to a coronal position of the bone peak to the implant-abutment interface. Then, the software was changed to the superimposed pre-operative image so that the pre-operative vertical buccal bone level could be assessed while all reference lines remained. Finally, vertical buccal bone loss was calculated by subtracting the one-year vertical buccal bone level from the pre-operative vertical buccal bone level.

### 2.6. Statistical Analysis

A sample size calculation was based on the primary study outcome (change in buccal soft tissue profile) and resulted in the inclusion of 30 patients per group (for details see Cosyn et al. [12]). 

SPSS Statistics 27 (SPSS Inc., Chicago, IL, USA) was used for data analysis. Five patients were randomly selected for duplicate bone measurements by another clinician. Inter-assessor reliability was assessed using intra-class correlation coefficient (ICC). Horizontal buccal bone loss at the different levels and vertical buccal bone loss were expressed as mean values, standard deviations and 95% confidence intervals. The paired samples *t*-test was applied to evaluate whether significant bone loss had occurred between the pre-operative situation and the one-year follow-up within each group. Bone loss at the one-year follow-up was compared between the groups using the independent samples *t*-test. The level of significance was set at 0.05.

## 3. Results

### 3.1. Patients

**Figure 3:**
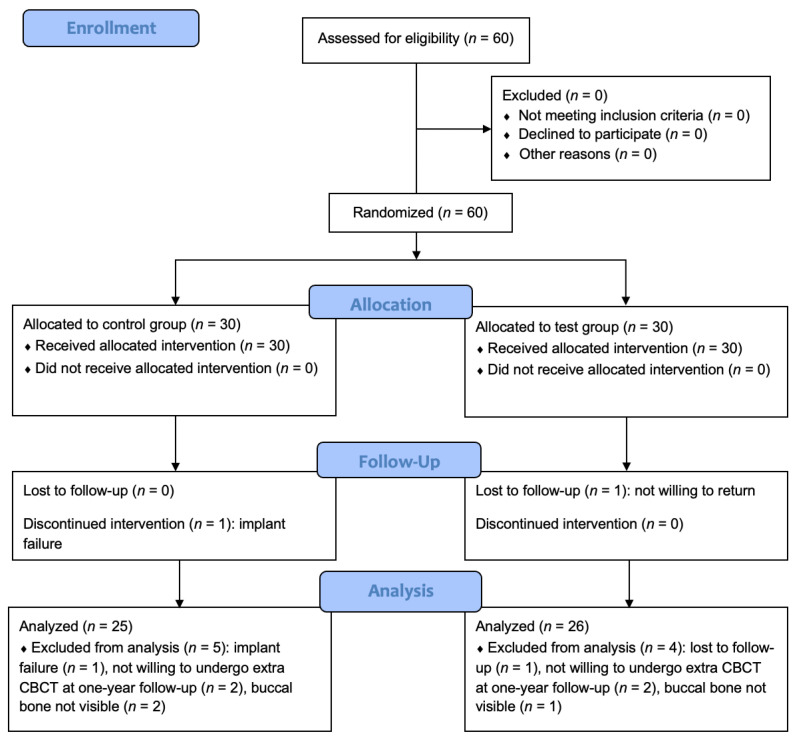
CONSORT flow diagram.

The CONSORT flow diagram is shown in Figure 3. Sixty patients were enrolled in this multi-centre RCT. The control group consisted of 15 males and 15 females with a mean age of 50.1 (SD 17.0) years. In the test group, 14 males and 16 females with a mean age of 48.2 (SD 16.3) years participated. Details on implant positions and parameters can be found in an earlier paper [12].

One implant in the control group was lost at the one week follow-up due to mobility. One patient in the test group was not willing to return for re-assessment at the one-year follow-up. Of the remaining 58 patients, four patients (two in the control group and two in the test group) were not willing to undergo an extra CBCT at the one-year follow-up. Three patients (two in the control group and one in the test group) could not be analyzed for buccal bone loss because buccal bone was not visible on CBCT. Thus, out of the original sample of 60 patients, 51 patients (25 in the control group and 26 in the test group) could be analyzed for buccal bone loss.

### 3.2. Horizontal Buccal Bone Loss

Inter-assessor reliability on bone measurements was excellent given an ICC of 0.962 (*p* < 0.001) (95% CI: 0.929–0.980). 

**Figure 4:**
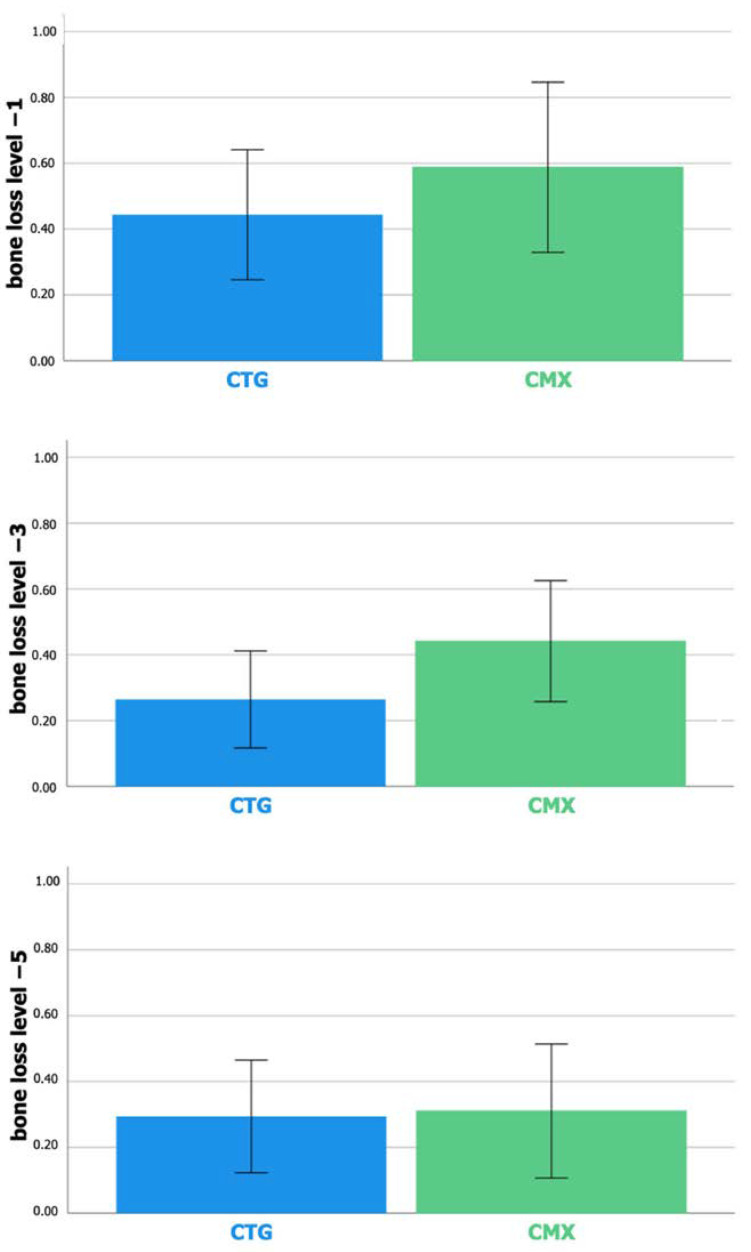
Bar charts representing mean horizontal buccal bone loss at 1 mm, 3 mm and 5 mm apical to the implant-abutment interface, respectively. Error bars: 95% confidence interval.

Figure 4 shows the results on horizontal buccal bone loss at 1 mm, 3 mm and 5 mm apical to the implant-abutment interface, respectively. Irrespective of the level and group, statistically significant horizontal bone loss occurred. 

At level −1 mm, most horizontal resorption was found pointing to 0.44 mm in the control group and 0.59 mm in the test group. The difference of 0.14 mm (95% CI: −0.17–0.46) between the groups was not statistically significant (*p* = 0.366). At level −3 mm, horizontal buccal bone loss amounted to 0.26 mm in the control group and 0.44 mm in the test group. The difference of 0.18 mm (95% CI: −0.05–0.40) between the groups was not statistically significant (*p* = 0.128). At level −5 mm, horizontal buccal bone loss amounted to 0.29 mm in the control group and 0.31 mm in the test group. The difference of 0.02 mm (95% CI: −0.24–0.28) between the groups was not statistically significant (*p* = 0.899).

### 3.3. Vertical Buccal Bone Loss

**Figure 5:**
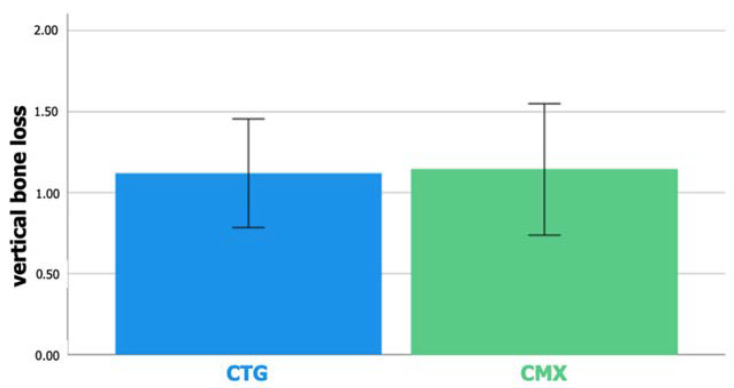
Bar chart representing mean vertical buccal bone loss. Error bars: 95% confidence interval.

Figure 5 shows the results on vertical buccal bone loss. This amounted to 1.12 mm in the control group and 1.14 mm in the test group. The difference of 0.02 mm (95% CI: −0.53–0.49) between the groups was not statistically significant (*p* = 0.926).

## 4. Discussion

In this study, the safety of buccal soft tissue augmentation using CMX was evaluated because recent findings had indicated deeper pockets, more marginal bone loss and more midfacial recession at 3 months follow-up when compared to CTG [12]. Even at a one-year follow-up, CMX treated sites still showed more marginal bone loss than CTG treated sites [11]. CMX and CTG were placed in contact with the buccal bone wall, as they had been applied following full thickness mucoperiosteal flap elevation. Given this, analyzing buccal bone loss became an evident method to assess their safety.

Both surgical procedures induced buccal bone loss. As shown in a recent systematic review, flap elevation by itself induces bone loss [23]. What the additional impact of a grafting material in proximity of the bone is, cannot be elucidated in this study given the lack of a control group without soft tissue augmentation. On the other hand, horizontal bone loss was limited ranging from 0.26 mm to 0.59 mm depending on the level and the group. Hence, the possible impact of a grafting material on bone loss, if any, could not have been clinically relevant.

A positive finding was that CMX did not induce more buccal bone loss than CTG at any level (*p* ≥ 0.128). Therefore, the null hypothesis was accepted. In a preclinical study, CMX showed moderate degradation of the matrix network and slight to moderate infiltration with inflammatory cells during the first 2 months [17]. CTG treated sites demonstrated complete degradation of the graft with no inflammation at 2 months. This was confirmed by human histology [24]. The fact that more inflammation may be expected around CMX than around CTG during the early phases of healing may explain deeper pockets, more marginal bone loss and more midfacial recession we observed at 3 months follow-up [12]. However, after 3 months, degradation and remodeling seem to take over [17,25]. This may explain the lack of a statistically significant difference between CMX and CTG treated sites in terms of buccal bone loss at one-year follow-up. One should take into account that the present study may have been underpowered to identify a mean difference of 0.14 mm (level –1 mm) and 0.18 mm (level –3 mm) as statistically significant, because the sample size calculation was based on change in buccal soft tissue profile and not on buccal bone loss. On the other hand, this may be an academic discussion because a mean difference of 0.14 mm or 0.18 mm may not be considered clinically relevant.

Vertical buccal bone loss was also observed in both groups, pointing to 1.12 mm in the control group and 1.14 mm in the test group. Again, the difference of 0.02 mm was not statistically significant (*p* = 0.926). Vertical bone loss mainly reflects marginal bone remodeling, which seems relatively high in this study. On the other hand, one should keep in mind that the included cases had a narrow alveolar ridge leaving in general a thin layer of bone at the buccal aspect of the implant. Such thin bone may be prone to resorption. In addition, all implants had a turned collar of 0.8 mm, which may also induce extra remodeling [26].

The following limitations must be taken into account for an appropriate interpretation of the results of the present study. First, nine out of 60 treated patients (15%) could not be analyzed for a variety of reasons. On a positive note, there was no selective loss to follow-up, and only very few cases in either group demonstrated no visible buccal bone. Second, the sample size calculation was based on change in buccal soft tissue profile, not on buccal bone loss. Hence, the present study may have been underpowered. Third, it is sometimes difficult to assess the true buccal bone surface on CBCT when an implant has been installed due to scattering and beam hardening. However, excellent inter-assessor reliability on bone measurements was found.

## 5. Conclusions

In the short term, soft tissue augmentation with CTG or CMX results in limited buccal bone loss. CMX is a safe alternative to CTG. Longer follow-up is needed to assess the impact of soft tissue augmentation on buccal bone.

## Data Availability

The data presented in this study are available in Supplementary Materials.

## Supplementary Materials

The following supporting information can be downloaded at: https://www.mdpi.com/article/10.3390/jcm12082977/s1, Table S1: Dataset_FibroGide_CBCT.
